# Systematic Review of Gallbladder Disease in Type 1 Diabetes Mellitus: Evidence on the Association With Cholecystitis

**DOI:** 10.7759/cureus.93416

**Published:** 2025-09-28

**Authors:** George Horton

**Affiliations:** 1 Diabetes and Endocrinology, University Hospitals of North Midlands, Stoke-on-Trent, GBR

**Keywords:** biliary pathology, cholecystitis, gallbladder disease, systematic review, type 1 diabetes mellitus

## Abstract

Cholecystitis is a common gastrointestinal condition strongly associated with type 2 diabetes mellitus (T2DM). However, the relationship between type 1 diabetes mellitus (T1DM) and cholecystitis is poorly defined. To date, no prior systematic review has specifically evaluated this association. This review aimed to evaluate whether T1DM is associated with cholecystitis, gallstones, or broader gallbladder disease and to identify gaps in the literature. A systematic search of Embase, Ovid Emcare, Ovid MEDLINE, and the NHS Knowledge and Library Hub was conducted (last search April 2025). Eligible studies were observational, comparing people with T1DM to nondiabetic or T2DM populations. Case reports, reviews, and studies not distinguishing between diabetes types were excluded. Risk of bias was assessed using the Newcastle-Ottawa Scale for cohort studies and the Joanna Briggs Institute checklist for cross-sectional studies. Due to heterogeneity, a qualitative synthesis was performed. Four studies met the inclusion criteria: two cross-sectional, one retrospective cohort, and one database analysis. Evidence was inconsistent. One cohort study reported a reduced gallstone risk in young adults with T1DM (adjusted hazard ratio: 0.48, 95% CI: 0.25-0.92). A pediatric study found no gallstones among 105 children with T1DM. Broader gallbladder disease outcomes showed no consistent associations after adjustment for confounders. Notably, no study specifically evaluated acute cholecystitis. Current evidence does not demonstrate a clinically significant association between T1DM and acute cholecystitis. The small number and heterogeneity of available studies limit the ability to draw firm conclusions. Further large-scale, prospective research using standardized diagnostic criteria is needed.

## Introduction and background

Cholecystitis, an inflammatory condition of the gallbladder, is a common cause of abdominal pain and hospitalization [1]. Its association with T2DM is well established, with several systematic reviews confirming an increased risk of gallstones and gallbladder disease in this population [2]. However, the relationship between T1DM and cholecystitis remains poorly defined.

Several mechanisms could be suggested to explain a possible link between T1DM and gallbladder disease. These include autonomic neuropathy that could impair gallbladder contraction, while immune-mediated processes or changes in gut motility might also play a role. In T2DM, altered bile composition and impaired gallbladder emptying are well described [3], but it is unclear whether these same processes occur in T1DM. Moreover, since acute cholecystitis is usually triggered by gallstones, the still-uncertain prevalence of gallstones in people with T1DM makes it difficult to establish a clear causal pathway.

Despite decades of research into biliary disease in diabetes, no systematic review has specifically examined whether T1DM confers a distinct risk for cholecystitis or other gallbladder pathology. This review, therefore, aimed to synthesize the available evidence and identify key gaps in the literature.

## Review

Methods

A systematic review was conducted in accordance with the Preferred Reporting Items for Systematic reviews and Meta-Analyses (PRISMA) 2020 guidelines [4]. Searches were carried out in Embase, Ovid Emcare, Ovid MEDLINE, and the NHS Knowledge and Library Hub using combinations of terms such as “Type 1 Diabetes Mellitus”, “T1DM”, “Insulin-dependent diabetes”, and “Autoimmune diabetes” along with “Cholecystitis”, “Gallbladder inflammation”, and “Gallbladder disease”. PubMed was excluded from the final search strategy as it yielded no additional eligible results. Searches were last conducted in April 2025. The full search strategy is detailed in Table 1.

**Table 1:** Full search strategy

Database	Search strategy	Results
Embase/Ovid Emcare/Ovid MEDLINE	1. type 1 diabet*.ti,ab.	177,571
	2. insulin dependent diabet*.ti,ab.	60,175
	3. (T1DM or T1D).ti,ab.	66,051
	4. autoimmune diabet*.ti,ab.	10,142
	5. 1 or 2 or 3 or 4	247,280
	6. cholecystitis.ti,ab.	44,456
	7. (gallbladder adj2 (disease* or inflam*)).ti,ab.	7,982
	8. 6 or 7	50,069
	9. (risk* or link* or association* or incidence* or predisp* or relationship* or prevalence* or complication*).ti,ab.	23,468,648
	10. 5 and 8 and 9	58
	11. remove duplicates from 10	39
	12. limit 11 to English language	36
	13. limit 12 to humans	35
NHS Knowledge and Library Hub	TI ( type 1 diabetes OR t1d OR insulin-dependent diabetes ) OR AB ( type 1 diabetes OR t1d OR insulin-dependent diabetes ) AND TI ( cholecystitis OR gall bladder inflammation OR gall bladder disease ) OR AB ( cholecystitis OR gall bladder inflammation OR gall bladder disease )	-

Studies were included if they were observational (cohort, case-control, or cross-sectional) and reported data on cholecystitis or gallbladder disease in people with type 1 diabetes mellitus (T1DM), with comparisons made either to nondiabetic individuals or to individuals with type 2 diabetes mellitus (T2DM). Studies were eligible if they provided stratified results for T1DM or clearly identified T1DM as a separate exposure group.

**Figure 1:**
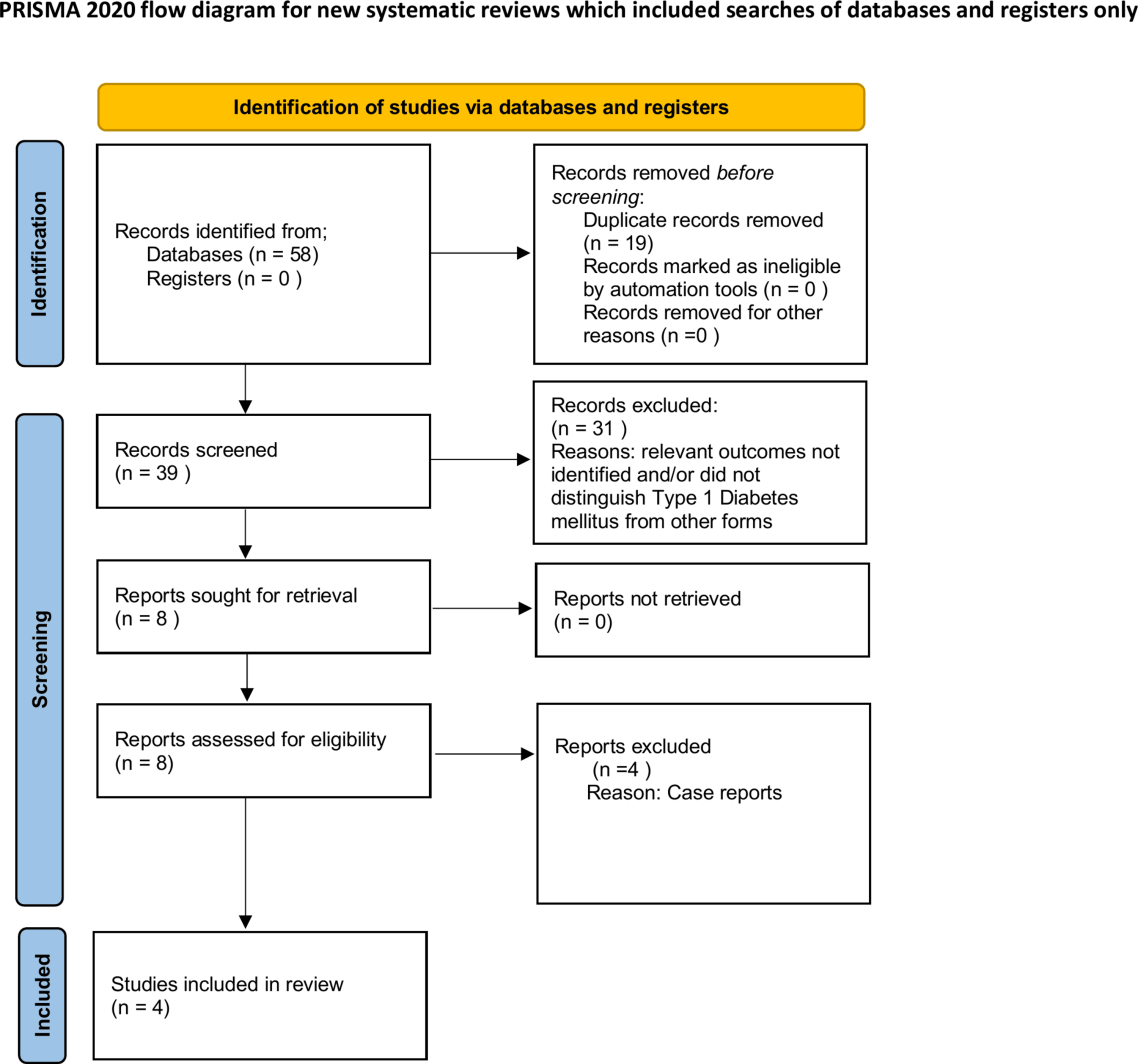
PRISMA 2020 flow diagram Flow diagram outlining the study selection process. Databases searched: Emcare, Embase, MEDLINE, and the NHS Knowledge and Library Hub. A total of 58 records were identified through database searches. After the removal of 19 duplicates, 39 records were screened. Of these, 31 were excluded because relevant outcomes were not identified and/or the studies did not distinguish T1DM from other forms. Eight full-text articles were assessed for eligibility, of which four were excluded as case reports. Four studies were included in the qualitative synthesis. PRISMA, Preferred Reporting Items for Systematic reviews and Meta-Analyses

Exclusion criteria included case reports, reviews, editorials, and studies that did not distinguish between T1DM and T2DM populations. The study selection process is illustrated in the PRISMA 2020 flow diagram (Figure 1) [5]. At the screening stage, 31 records were excluded because they did not report relevant outcomes and/or did not distinguish T1DM from other forms. At the full-text review stage, four articles were excluded as case reports. Data were extracted on study design, sample size, population characteristics, outcome definitions, and effect estimates. Study quality was assessed using the Newcastle-Ottawa Scale [6] for cohort studies and the Joanna Briggs Institute checklist [7] for cross-sectional studies. Studies were assessed according to the domains specified in each tool, including selection, comparability, and outcome measurement. The full scoring and justifications are provided in Table 2.

**Table 2:** Quality and risk of bias assessment with justifications

Study	Design	Tool used	Score	Risk of bias	Justification
Chen et al. [8] (2018)	Cohort	Newcastle-Ottawa Scale [6]	9/9	Low	Controlled for key confounders using adjusted hazard ratios. Based on a large national cohort with adequate follow-up via claims data.
Al-Hussaini et al. [9] (2013)	Cross-sectional	Joanna Briggs Institute checklist [7]	6/8	Moderate	No adjustment for confounders; descriptive analysis only. Study lacked a control group, limiting the ability to assess comparative risk.
Chapman et al. [10] (1996)	Cross-sectional	JBI	8/8	Low	Confounders identified and addressed using stepwise logistic regression. Outcomes measured objectively.
Ali and Adetunji [11] (2018)	Cross-sectional (retrospective database study)	JBI	8/8	Low	Used a large administrative database. Multivariable regression was applied to adjust for confounders, though outcome identification relied on diagnostic coding, introducing potential misclassification bias.

Results

A total of four studies met the inclusion criteria: two cross-sectional studies, one retrospective cohort study, and one large-scale database analysis. Owing to heterogeneity in outcome definitions and study designs, findings are presented by outcome type. Key characteristics of the included studies are summarized in Table 3, and the study selection process is illustrated in the PRISMA 2020 flow diagram (Figure 1) [5].

**Table 3:** Study characteristics

Study	Country	Study design	Population	Comparator	Outcome	Key findings
Chen et al. (2018) [8]	Taiwan	Retrospective cohort	Young adults with T1DM	General population	Gallstones	Reduced risk (adjusted hazard ratio: 0.48, 95% CI: 0.25-0.92)
Al-Hussaini et al. (2013) [9]	Saudi Arabia	Cross-sectional	105 pediatric T1DM patients	No control group	Gallstones	No gallstones detected by ultrasound
Chapman et al. (1996) [10]	New Zealand	Cross-sectional	Adults with T1DM and T2DM	Nondiabetics	Gallbladder disease	Higher prevalence in females with T1DM (unadjusted), but not significant after adjustment
Ali and Adetunji (2018) [11]	USA	Retrospective database study	T1DM and T2DM patients	Nondiabetics and T2DM	Biliary diseases (not cholecystitis-specific)	Lower rates than T2DM but higher than nondiabetics; cholecystitis not isolated as an outcome

With regard to gallstones, two studies provided relevant data. Chen et al. (2018) [8] conducted a large retrospective cohort study using Taiwan’s National Health Insurance Research Database and reported a significantly reduced risk of gallstone disease among young adults with T1DM (adjusted hazard ratio: 0.48; 95% CI: 0.25-0.92). This protective association remained significant after adjustment for age, sex, and comorbidities, suggesting a potential biological link in this demographic. In contrast, Al-Hussaini et al. (2013) [9] performed a cross-sectional study involving 105 pediatric patients with T1DM in Saudi Arabia and found no cases of gallstones on abdominal ultrasound. However, the absence of a nondiabetic control group and the lack of adjustment for potential confounders limit the interpretability of these findings. Together, these studies suggest that gallstone prevalence in T1DM may vary by age group, with some evidence of reduced risk in younger adults but inconclusive findings in children.

Two additional studies evaluated broader gallbladder disease outcomes without isolating gallstones or cholecystitis. Chapman et al. (1996) [10] conducted a cross-sectional study in New Zealand comparing diabetic and nondiabetic individuals. Among women, the unadjusted prevalence of gallbladder disease was higher in those with T1DM (36.3% vs. 23.1%), although this difference was not statistically significant once adjustments were made for age and BMI. Diagnoses were confirmed using ultrasound, and confounders were addressed with stepwise regression analysis. Ali et al. (2018) [11] used a large US administrative claims database to evaluate biliary disease, including gallstones and cholecystitis, in diabetic populations. Their analysis showed that patients with T1DM had lower rates of gallbladder disease compared with those with T2DM, but rates were still higher than in nondiabetic individuals. The study did not specifically evaluate cholecystitis as a separate outcome and relied on diagnostic coding, introducing the possibility of misclassification bias.

Taken together, these four studies provide limited and inconsistent evidence regarding the association between T1DM and gallbladder disease. While one high-quality cohort study suggested a reduced risk of gallstones in young adults, a pediatric study found no cases, and other studies addressing broader gallbladder outcomes reported no consistent associations after adjustment for confounding factors. Importantly, no study specifically evaluated acute cholecystitis as an isolated outcome. Based on this comprehensive literature search, no direct evidence currently exists to support an association between T1DM and cholecystitis.

Discussion

This is the first systematic review to specifically evaluate the association between T1DM and cholecystitis. Across the four eligible studies, findings were limited and inconsistent. While Chen et al. reported a protective effect of T1DM in younger adults [8], other studies did not confirm this, and no direct evidence was found linking T1DM with acute cholecystitis.

The lack of consistency may reflect differences in study populations, design, and outcome definitions. Pediatric data are scarce, and adult studies vary in whether gallstones or broader gallbladder disease was measured. Adjustment for confounders such as age, BMI, and comorbidities was not uniform. Furthermore, reliance on administrative coding in large-scale datasets introduces potential misclassification.

These findings contrast with T2DM, where systematic reviews and meta-analyses consistently demonstrate an increased risk of gallstones and gallbladder disease [2]. Mechanisms proposed in T2DM include impaired gallbladder motility and altered bile composition [3]. These may not fully apply to T1DM; however, autonomic neuropathy, immune dysregulation, and glycemic control could plausibly contribute to biliary risk [12].

The wider literature reinforces this contrast. Large population-based reviews of gallstone epidemiology, such as Stinton and Shaffer [12], identify diabetes as a consistent risk factor for gallstones at the population level. Similarly, Nervi et al. demonstrated a strong association between insulin resistance and gallbladder disease, suggesting that metabolic dysfunction is a central contributor to biliary pathology [13]. While this is well established in T2DM, the distinct metabolic profile of T1DM has been insufficiently studied in this context.

Taken together, current evidence does not support a clinically significant association between T1DM and acute cholecystitis. Importantly, this review highlights a major gap in the literature. Whether T1DM independently influences gallbladder disease risk remains under-researched. Future work should prioritize prospective, population-based studies using standardized diagnostic criteria, with subgroup analyses by age, sex, and glycemic control, to clarify whether T1DM confers a distinct risk profile.

Limitations

This review is limited by the small number of eligible studies and their heterogeneity in design, populations, and outcome definitions. Several studies used retrospective or descriptive methods, and adjustment for confounders was inconsistent. The absence of standardized diagnostic criteria (e.g., Tokyo Guidelines) [14], lack of prospective data, and potential publication bias further limit reliability.

Only English-language studies were included, which may have excluded relevant non-English publications. This review was not registered in PROSPERO, which could have enhanced transparency. The small number of studies precluded meta-analysis, and certainty assessment was not applicable.

Strengths of this review include its novelty as the first systematic review specifically addressing the association between T1DM and cholecystitis and the use of a structured PRISMA 2020 methodology with transparent eligibility criteria and quality assessment.

## Conclusions

Current evidence on the association between T1DM and cholecystitis is inconclusive. While one cohort study suggested a reduced risk in younger adults, overall the evidence base remains limited by small study numbers, varied designs, and inconsistent outcome definitions. Further large-scale, prospective studies using standardized diagnostic criteria are needed to clarify whether T1DM confers a distinct risk profile for cholecystitis or other gallbladder diseases. Overall, this review found no statistically significant evidence supporting a consistent association between T1DM and cholecystitis.
